# A qualitative analysis of free-text patient satisfaction responses in Care Response, a database of patient-reported outcome and experience measures

**DOI:** 10.1186/s12998-023-00528-7

**Published:** 2024-01-29

**Authors:** Kenneth J. Young, Helen C. Young, Jonathan Field

**Affiliations:** 1 University of Central Lancashire, Preston, UK; 2 Private Practice of Chiropractic, Walton-Le-Dale, UK; 3 University of Southampton, Southampton, UK

**Keywords:** Chiropractic, Patient-reported experience measures, Word frequency analysis, Content analysis, Patient satisfaction

## Abstract

**Background:**

Databases have become important tools in improving health care. Care Response is a database containing information on tens of thousands of chiropractic patients internationally. It has been collecting patient-reported outcomes and patient satisfaction information for more than 10 years. The purpose of this study was to contribute to the understanding of patient perceptions and priorities for chiropractic care by analysing free text entered into the patient reported experience measure (PREM) questionnaires within the Care Response system.

**Methods:**

There were two questions of interest on the PREM for this study. One requested information about “good points” patients perceived about patients’ care experience, and the other requested information on “improvements” that could make the experience better. We conducted a word frequency analysis using a word counting macro in Microsoft Word, then used those results as a starting point for a qualitative analysis. Data were collected on 30 May 2022.

**Results:**

The people who participated in the Care Response system often reported positive experiences with their chiropractors, including that they had reduced pain, improved function, and felt validated in their clinical condition. In addition, they appreciated having diagnostic and treatment procedures explained to them. They valued friendly, professional, and on-time service. The negative experiences were the opposite: being rushed through treatment, that the treatment was not worth the cost, or that they weren’t treated professionally, empathetically, or with respect for them as individuals. The most important themes that emerged under “good points” were satisfaction (with care), value (as a person), safety, comfort, and professionalism. Their opposites, dissatisfaction, lack of value, lack of safety, lack of comfort, and lack of professionalism emerged as the most important themes under “improvements”. We report some nuances of patient experience that have not previously been explored in the literature.

**Conclusions:**

Respondents seemed to value effective care provided in a safe, professional, friendly, and aesthetically pleasing environment. Chiropractors should note these priorities and engage with patients according to them. Education institutions should consider how good practice in these areas might be incorporated into curricula.

**Supplementary Information:**

The online version contains supplementary material available at 10.1186/s12998-023-00528-7.

## Background

Digital information management systems, including databases, have become important tools in understanding trends and correlations in health care by improving the collection and organisation of information. Access to information on demographics, diagnostics, therapeutic interventions, and outcomes has been enhanced through electronic storage and retrieval methods. Routine collection of such data has now become a commonplace in private as well as national health systems. The United Kingdom National Health Service (NHS) has promoted recent initiatives focusing on the most efficient use of such data for key goals including improving patient outcomes, increasing efficiency of health care delivery, and the development of new treatments [1].

Analysis of data collected from large cohorts of patients has the potential to generate insights into factors identifying effective therapeutic interventions as well as determining characteristics of subgroups of patients who respond to those interventions. Observational studies using large data sets have led to important public health discoveries through epidemiological analysis. For example, the Framingham study greatly improved the understanding of the role of blood pressure in disease [2].

The Care Response (CR) database collects patient-reported outcome measures (PROMs) and patient-reported experience measures (PREMs) internationally. The CR database was developed as an electronic system to help clinicians overcome some of the barriers to using PROMs regularly in clinical practice. Electronic PROM systems have been shown to simplify the collection and reporting of results as well as increase the completeness of returned assessments [3–5]. PROMs available on the CR system include the Bournemouth Questionnaire (BQ), Measure Your own Medical Outcome Profile (MYMOP), EQ-5D, patient global impression of change (PGIC), numerical pain scale, and Patient Reported Experience Measure (PREM, [6]). Clinicians opt into whichever of the PROMs they would prefer to use in their practice.

The CR database is currently provided to clinicians free of charge with a web-based interface. Care Response allows direct acquisition of information from patients, rather than relying on clinicians to administer PROMs and PREMs themselves during consultations. After obtaining informed consent, clinics register patients by adding their name, date of birth, email address and date of first appointment either using a’self-service’ link provided by the clinic or by clinic administrative staff. Once these fields are populated, the CR database generates a PROM questionnaire, based on the clinician’s preference, and this is provided to the patient usually via an automated email link or by the clinic on a PC or tablet device, or in paper form. Any of the available PROM/PREM instruments can be selected as a whole; questions cannot be selected from different PROMs and combined by a clinician. Multiple PROMs may be selected for use by any clinic. Subsequent PROM questionnaires are generated either at pre-set timed intervals or clinicians may manually request them. PROM questionnaires are scored, and the clinician is presented with collated results for an individual patient or group of patients in tabulated or graphic format. Data are available immediately and can be collated to allow for longitudinal assessments.

The CR database had 218,770 patients registered as of September 2022. It has been adopted in clinical practice across diverse settings and multiple countries. However, up to date information on the number of clinics or countries is not available, as the system is anonymised. There are also no data on the numbers of NHS versus private patients. Software coding to extract this information from the database to provide this could be developed but it does not exist currently.

Several studies based on Care Response data have been previously published [7–17]. Part of what the CR database collects is information on patients’ opinions on satisfaction with their chiropractic care. Patients are invited to answer PREM questionnaires a month after their first appointment with a clinic. In addition to specific questions relating to experiences with interactions with clinical and non-clinical personnel at the clinics there are two free text boxes that patients are offered if they opt to participate in the system. Those questions are (1) Is there anything particularly good about your chiropractic care? and (2) Is there anything that could be improved?

Surveys have shown that people who attend chiropractors are generally satisfied with the care that they receive [18–23]. Rowell and Polipnick [24] combined a survey with a semi-structured interview and reached a similar conclusion but noted that more factors influencing satisfaction are present than are measured in standard satisfaction outcome instruments. Offering people the opportunity to express themselves in their own words often results in rich data, that is, more detail and nuance than is usually available through surveys. Asking people open-ended, general questions, the way the CR system does, also allows people to set their own priorities for the topic(s) they would like to address. Free-text PREMs can help improve value-based care, provide deeper insights on patient experiences or on subpopulations [25–27]. They can also identify issues that closed-ended questions might not reveal and guide the development of new survey questions [28–30].

Our aim with this study was to better understand patient perceptions and priorities for chiropractic care. As yet, there has been no assessment of the patient satisfaction comments on the CR database. The purpose of this study was to conduct a qualitative analysis of the free text responses entered into the patient-reported experience section of the Care Response database.

## Methods

Before the start of data collection, the legal team at the University of Central Lancashire (UCLan) drew up data sharing agreements between UCLan and Clinical Transparency, Ltd, which holds the CR database. Ethical approval was obtained through the UCLan Research Ethics Committee, approval number HEALTH0287. Consent to participate is obtained by Care Response and includes the option of use for research by third parties. The search was not date-limited, seeking data from the full 10 years of the existence of Care Response. Data were collected on 30 May 2022. The researchers are all chiropractors in clinical practice, two have PhDs and experience in qualitative methods.

Because open-ended questions allow participants to express their views on the issues of most importance to them, we decided on an inductive approach to a qualitative content analysis using the steps described by Peterson–Lewis [31]. However, because of the size of the CR database, we anticipated receiving many thousands of responses and sought a way to manage the data, given limited time and resources and also to determine which themes were most important to participants. Sandelowski [32] noted that numbers are integral to qualitative research. Pattern recognition in data implies identifying recurring patterns and displaying information numerically can help avoid over- or underweighting data.

Thus, we devised a novel method to make sense of the data. The use of quantitative and qualitative approaches in combination may provide a better understanding of research problems and complex phenomena than either approach alone [33]. It has also been noted that mixing the two methods cancels out, somewhat, their corresponding weaknesses. Quantitative research, although reliable, is often criticized for the validity of its outcomes, and although qualitative research has good validity, there are issues of repeatability and generalisability [34].

Two authors first manually scanned the data to get an idea of scope and completeness. We then applied a word frequency analysis. The rationale was that we could then explore the more common themes first, to develop a sense of perspective on the data. We excluded common verbs, adverbs, articles and conjunctions, such as “is”, “very”, “the” and “and”. We included adjectives as these modifiers were deemed to denote special importance to whatever issue respondents were addressing. All the text from both the “good points” and “improvements” text boxes were copied separately into two separate Microsoft Word documents. Using a word counting macro, a list of all words in order of occurrence was generated for both sets of data. We also created two word clouds using a free, online tool at www.wordclouds.com to allow a visual representation of word usage for both the “good points” and “areas for improvement” responses. Without being definitive, we believed that this could help us to start developing a sense of respondents’ priorities.

Two authors independently searched for each of the 20 most common words in the responses. The 20 most common words were used as a starting point to interrogate the data. They gave a focus for the initial development of codes and themes, representing areas commonly invoked by participants. We then applied a process similar to thematic analysis, However, given that the relatively short answers in the free-text boxes provided by Care Response did not allow for the richness of data often analysed in thematic analysis, we classed our process as content analysis rather than thematic analysis. Nonetheless, we applied 6 steps as described by Braun and Clarke [35]: familiarisation with the data; initial coding; searching for themes; reviewing themes; defining and naming themes and resolving any disagreements by discussion; and finally producing the report using illustrative quotations. Although our method did not ensure saturation of information, we believe that it is useful in revealing the priorities of these participants regarding their experiences with chiropractic care. We used an iterative process of coding, combining responses that denoted similar concepts [36]. The coding process was data driven rather than theory driven. We then searched for themes, into which the coded data extracts could be placed. Two authors then reviewed and revised the themes, developing 1st and 2nd order themes.

## Results

The data extract from CR servers was returned in an Excel spreadsheet with 21,667 rows, each representing one patient. The date range of the entries was 09 July 2012 to 09 May 2022. There were 8624 people who entered text in the “good points” box, for a response rate of 40% of patients who entered any information in the CR system. For the “improvement” box, 3202 people responded, for a response rate of 15%. People could enter text in both boxes; they were not mutually exclusive. The demographics of respondents can be found in Table 1.

**Table 1:** Demographics

Gender		%	Ethnicity		%	Age (years)	Median number of times seen by practitioner
Female	12,035	55.5%	White	20,766	95.9%	Median: 48 Range: 2–113 Inter-quartile range: 24	3 Interquartile range: 2
Male	8902	41.1%	Asian	314	1.4%		
Unknown	730	3.4%	Other	180	0.8%		
			Chinese	55	0.3%		
			Mixed	214	1.0		
			Black	138	0.6%		

**Fig. 1:**
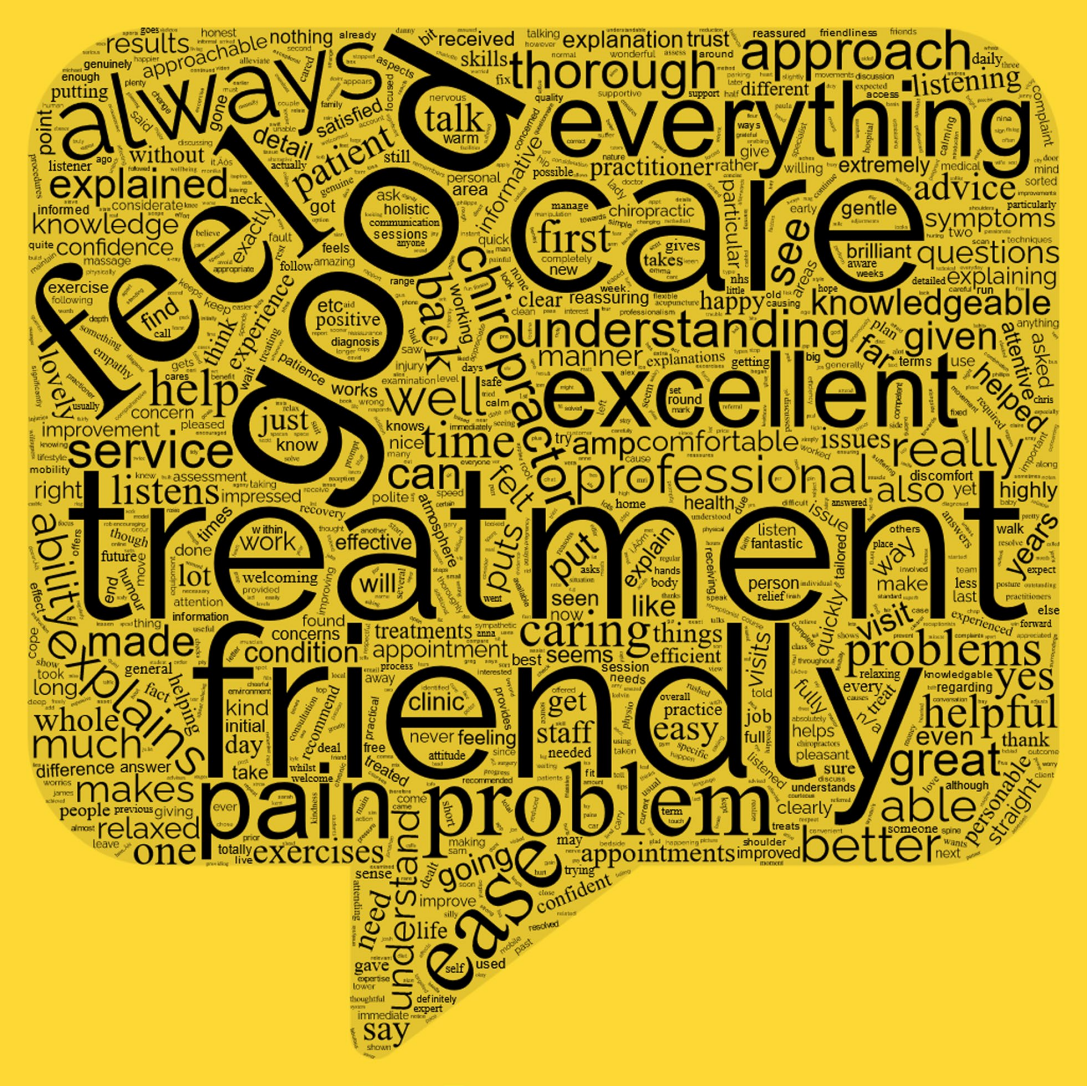
"Good points" word cloud

**Fig. 2:**
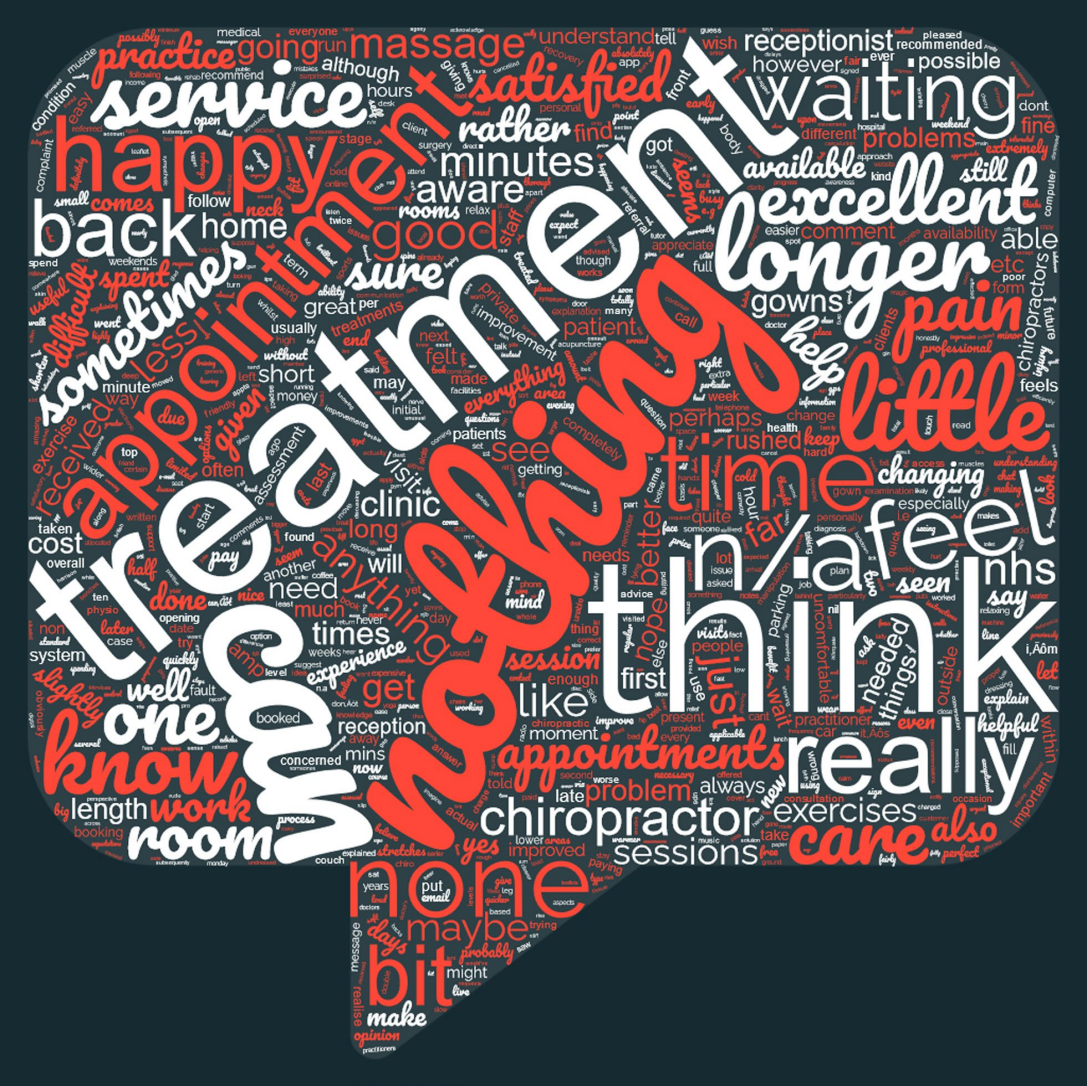
"Improvements" word cloud

Please see Figs. 1 and 2 for word clouds of all responses for each category; these are a visual representation of the rate of occurrence of words. The text revealed up to 9 separate codes for each word. Please see Table 2 and 3 (at end of paper) for the initial codes in which the words were used. Please see Additional File 1 for full quotes representing an example of each code for each word. Please see Table 4 for the 1st and 2nd order themes and consolidated codes developed from the initial codes.

**Table 2:** Prevalence and initial codes for top 20 most frequent words in “good points” free text box

Word	Number of cells with word	Code 1	Code 2	Code 3	Code 4	Code 5	Code 6	Code 7	Code 8	Code 9
Treatment	1227	Effective/happy/satisfied with/ impressed	Prompt	Explains/articulates	Confident about	Relaxed/not rushed	Professional	Diverse range of		
Feel (-ing)	1059	Listened to/asked how I’m feeling	Comfortable/welcome/safe/at ease/in control	Patient is important/valued	[Made me feel] positive/encouraged	Better [less pain, more capable]	Confidence/trust [in the chiropractor]	Problems explained		
Good	1048	All round	Advice	Information/explanations/communication/conversations	Appointment times	Treatment/approach	Understanding/knowledge	Reception staff	Sense of humour	Listener/bedside manner
Problem(s)	996	Resolved/Reduced	Explained	Listens to/asks about/shows concern	Understands	Diagnosed/gets to the root				
Friendly	988	Unspecified	Chiropractor	Atmosphere/Clinic/Service	Staff/team					
Care	867	Chiropractor expresses	Satisfied with/can’t fault	Explains [treatment]	Feel cared for	Professional	Personalised/remembers me			
Chiropractor	845	Friendly	Knowledgeable/Informative	Listens/attentive	Professional/trust/honesty	Concerned/empathetic/caring/kind	Good/excellent/brilliant	Thorough	Gentle	
Ease(s)	828	Makes [me] feel at	The pain/problem							
Excellent	758	Chiropractor	Care/Treatment	All round/unspecified/clinic	Service	Staff	Listener	Explanations	Advice	Results
Professional	717	Unspecified	Chiropractor	Reception staff	Treatment/Service/approach	Attitude/manner				
Pain	601	Resolved/helped/reduced	Explained	Understood						
Caring	546	Unspecified	Chiropractor	Staff						
Time(s)	552	Takes time to listen/explain	Convenient appointment	Prompt/run on time						
Understanding	499	Unspecified	My needs/problem/activities	Chiropractor	Receptionist/staff was					
Thorough	480	History/Exam	Explanation	Unspecified/everything						
Always	477	Listens	Explains problem/informs	Feel at ease/welcome/	Professional	Feel better after appointment/works	On time	Friendly/courteous/polite		
Back	401	Made feel better	Explained problem							
Everything	397	Explains [treatment/exam]	About the clinic/experience	Listens to/Understands	Checks/examines/treats					
Service	358	Excellent/good	Personal	Friendly/caring/relaxing	Professional	Prompt				
Explains	354	Symptoms/problem/pain	Treatment/procedures	Unspecified/everything

**Table 3:** Prevalence and initial codes for top 20 most frequent words in “improvements” free text box

Word	Number of cells with word	Code 1	Code 2	Code 3	Code 4	Code 5	Code 6
Nothing/anything	642	Unspecified	At this time	Can/can’t think of			
Appointment(s)/session(s)	616	Longer treatment times	Different times (earlier, later, weekends, emergency)	Shorter waiting times	Would like reminder for	Would like on NHS	
Think	579	Nothing I can think of/can’t think of anything	More time for appointment	More attention to specific/personal needs			
Can/Can’t	541	Nothing I can think of	Can’t think of anything				
Time(s)/minutes	645	Longer appointment/treatment	Shorter waiting				
N/A/none	456	Unspecified					
Treatment	440	Better or written explanation of plan	High cost for	Longer time	No, satisfied with	Should be available on NHS	Less chat, more treatment/hands-on
Happy/good/excellent/satisfied	410	Satisfied					
Longer	187	Appointment/treatment					
Feel	182	Satisfied	Rushed/time could be longer	Explanations could be better	Facilities could be better	Would like more soft tissue work	Exposed (in gown or just underwear)
Chiropractor	163	Too short time with	Too long time waiting	Lack empathy/doesn’t smile	Could explain condition/treatment better		
Little or bit	148/117	More treatment time/feel rushed	Less waiting time	Facilities could be better	More information on care		
Service(s)	136	Satisfied with					
Wait(-ing)	129	Time too long (in reception or treatment room)	Better facilities in room (size, furniture, music, heat, etc.)	Time to first appointment	Lack of privacy		
Room	115	Temperature	Comfort issues: room size, hooks for clothes, type of furniture	Lack privacy/sound insulation	Waiting times	Aesthetic issues: décor, drinks, type of music, age/condition of facilities	
Exercise(s)	104	Would have liked	Clearer description or written instructions				
Clinic	100	Happy with	Better facilities (e.g. furniture, décor, cleanliness, parking, heat)	More availability for appointments	Busy, rushed		
Back	99	Wide variety of statements related to contexts already noted					
Massage (soft tissue work)	63	Would like incorporated into treatment					
Gown(s)	61	Unsatisfied with size, fit, fasteners	Uncomfortable/embarrassed being seen by others in a gown	Prefer to be told what kind of clothing to wear to avoid gown

**Table 4:** 1st and 2nd order themes and codes

Thematic area	1st order themes	2nd order themes	Consolidated codes
Good points	Satisfaction	Satisfied with care	Satisfied with treatment
			Experienced reduced pain
			Experienced improved function
			Felt confidence in their care
		Satisfied with staff	Felt confidence in practitioner
			Felt confidence in staff
		Unspecified satisfaction	
	Felt valued as a person	Good logistical support	Convenient appointment times
			Convenient location
			Prompt treatment/short waiting time
		Good emotional support	Felt listened to
			Empathy/caring expressed
			Personal needs understood/attended to
			Made to feel welcome
			Made to feel at ease
	Comfort	Physical comfort	Gentle treatment
		Mental comfort	Friendliness
			Courteous staff
			Relaxed/not rushed
			Practitioner/staff sense of humour
	Professionalism	Professional communication	Explained problem
			Explained diagnostic procedures
			Explained treatment
		Procedural professionalism	Thorough exam
			Thorough treatment
			Useful self-care plan
		Unspecified professionalism	
Areas for improvement	None		
	Dissatisfaction	Dissatisfaction with care	Wanted more home care/advice/exercise
			Wanted more soft tissue work
		Dissatisfaction with cost	Felt value lacking for money spent
			Would like chiropractic on the NHS
	Did not feel valued as a person	Poor logistical support	Appointment times not convenient
			Waiting times too long
	Lack of safety	Felt clothing was inappropriate for situation	Felt exposed in gown
			Felt exposed wearing underwear only for treatment
		Lack of audio privacy	Overheard personal details discussed by reception staff
			Overheard patients in other rooms
		Lack of cleanliness	
	Lack of comfort	Poor logistics	Lack of car parking
			Poor directions to clinic
		Poor infrastructure	Poor temperature control (too hot/too cold)
			Dilapidated/inappropriate structure/furniture
			Lack of facilities for less abled: stairs-only access, low chairs difficult to rise from
		Aesthetic/ancillary issues	Did not like décor
			Would like drinks/better selection of drinks
			Did not like type or volume of music played in reception
		Treatment attire	Difficult fasteners on gowns
			Sizes too big/too small
	Lack of professionalism	Poor professional communication	Lack of clear explanation of procedures
			Lack of written explanation of home care
			Lack of complete history
			Lack of empathy
		Poor professional procedures	Appointments too short

Although we attempted to develop discrete themes, some responses, especially longer ones, involved multiple codes and more than one theme. For instance, one response included the code, “waiting times too long” was classified under the 2nd order theme “poor logistical support” and 1st order theme “did not feel valued as a person”, but also mentioned a short appointment time (code), under “poor professional procedures” (2nd order), under the 1st order “lack of professionalism”. *“Waiting time is too long, up to 45 min one day! The whole appointment is rushed.”* In a few instances, we quoted the relevant parts of the entire response under the different categories below rather than the entire response if it was lengthy. All quotes are copied and pasted verbatim, so may include spelling/grammar errors.

### Results by theme with supporting quotes

Each quote is from a different participant under any given theme. However, any participant may be quoted in more than one theme.

#### Good points: general

Generally, the respondents in this study reported a positive perception of their chiropractic experiences: *“I think the care and treatment is very good”; “My practitioner has been exceptionally good and was spot on with diagnosis & treatment”.*

### Satisfaction

#### Satisfied with care

Participants wrote about experiencing reduced pain and improved function: *“The pain generally eases after a treatment”; “enabling good movement shortly after treatment”; “Yes it got me back playing badminton again.”*

#### Satisfied with staff

Satisfaction with staff was also reported: *“Very friendly staff. Nice waiting room—new magazines. Have confidence in Chiropractor”; “The chiropractor has a reassuring, confident and competent manner which helps you to feel relaxed. He is also highly skilful and has made my recovery very swift and eliminated the pain.”; “the whole process is operated in a professional and friendly manner. All the staff are eager to assist.”*

#### Satisfaction: unspecified

There were also responses indicating unspecified satisfaction. Unspecified meant that no further information about the use of a term was available. So, we created a second order theme for these responses: *“I was very satisfied with the services offered.”; “Satisfied”; “It 'IS' very good and I am 'VERY' satisfied with everything.”*

### Felt valued as a person

#### Felt valued as a person: good logistical support

Participants indicated that feeling valued as a person was important to a positive experience with care. Logistical support was one factor: *“ability to get an appointment at a convenient time with little notice”; “Have always been able to make appointment at a convenient time for myself. Have confidence in chiropractor.”; “Location is convenient, opening hours are very flexible”.*

#### Felt valued as a person: good emotional support

Emotional support was also a factor in feeling valued: *“I felt my chiropractor was very sympathetic and understood the frustration by back pain was creating for me. As well as the treatment I received I was given exercises to perform and shown exactly how to them. I have been very impressed with the treatment I received and believe my recovery has been swifter as a result.”; “I feel as though she really cares about the problems I have, and confident that she can help.”; “Put me at ease, good explaination of the problem, reduced worry, listened to my symtons/concerns—very proffesional.”; “She is able to put me at ease, so I feel comfortable and confident in her treatment.”; “Brilliant always friendly and makes me feel welcome and as comfortable as she can.”*

### Comfort

#### Physical comfort

Physical comfort was expressed regarding the treatment experience: *“It is a gentle treatment especially the first time when I couldn't walk properly and was in a lot of pain.”; “very approachable and understanding! Made things easy to understand and was gentle when giving my care”.*

#### Mental comfort

Mental comfort derived from experiencing friendly, courteous staff, from not feeling rushed, and from a demonstrated sense of humour: *“relaxed and friendly atmosphere”; “My chiropractor is down to earth, easily approachable and patient. She puts me at ease, whilst, at the same time, maintaining a high level of professionalism.”; “I feel very comfortable with my chiropractor. This is important to me. She puts me at ease. I had never visited chiropractors or osteopaths *etc. *before & I was very nervous. She explains everything to me very clearly and also listens. She adjusts my treatment after speaking to me about each visit.”; “Outstanding clinic and staff makes you feel so relaxed and not tensed would not use any other clinic.”; “He has made such an improvement to my well being, he cares and has a great sence of humour.”*

### Professionalism

#### Professional communication

Examples of professional communication included: *“Very well explained from what each step of the treatment is and the reasoning behind it.”; “The knowledge and understanding of the chiropractor and the diversity of the treatment—not only adjustments.”; “Excellent treatment and everything explained simply and thoroughly. Explains what future treatment might entail.” “First and foremost is the application of 'bedside manner', which enables an immediate rapport, which puts me at ease, and therefore able to relax. I understand the workings and functions of my body and so I am able to participate intelligently with my chiropractor. The adjustments are gentle with no violent or heavy handed moves. Discussion before, during and after treatment, is important in terms of understanding how progress is being made.”*

#### Procedural professionalism

Procedural professionalism was expressed in a variety of ways as well: *“Professional, a thorough assessment and treatment plan discussed”; “My practitioner is fantastic, I was very emotional at one of my appointments and was in quite a lot of pain. She was very caring and really took the time to explain to me how I could help myself and reduce my anxiety and pain levels. I was also given exercises to do, which have helped with the pain and with keeping mobile. When I called as my pain had increased, I was able to be seen on the next working day, which was great. Thorough and professional service.”*

#### Unspecified professionalism

Examples of unspecified professionalism included: *“very friendly but always professional”; “very professional”; “Total professionalism at all times”.*

### Areas for improvement

#### None

Many patients who chose to enter text in the “improvements” box that indicated they could think of none and were satisfied with their care. *“No, very impressed with my care and treatment”; “Have nothing to compare it with, but cannot see any areas needing improvement.”*

### Dissatisfaction

#### Dissatisfaction with care

We found dissatisfaction with care, mainly related to a lack of exercise advice or soft tissue work: *“Not really. I suppose I would like a bit more deep-tissue massage but I'm confident he would give it if he felt I needed it. I just like it as i’'s very relaxing!:)”; “Soft tissue treatment to speed the healing process of a pulled muscle.”*

#### Dissatisfaction with cost

Cost emerged as a negative factor, and a few advocated for having chiropractic subsidised or available on a national health service*. “The cost was very high considering my seoncd treatment was less than ten minutes. This was disproportionate.”; “Amount of time in the treatment. Should be available on nhs!”.*

### Did not feel valued as a person

Some respondents did not feel valued as people and thought that appointment times were inconvenient and waiting times too long. “*The clinic could be open later in the evenings, as many people can not get time off work.”; “I think improvements could be made in the waiting times, if I have an appointment for say 3.15 I would expect to be seen at that time, but usually have to wait at least 10 min longer.”*

### Lack of safety

#### Felt clothing was inappropriate for situation

We found several responses that we classified under safety issues. Some patients felt that the clothing they were asked to wear was inappropriate*: “Smaller gowns. As a size 8 women a big baggy gown does not feel safe and could fall off at any moment!”; “He made me feel uncomfortable standing in front of me, whist I was sitting in only my underwear and the gowns provided.”*

#### Lack of audio privacy

Audio privacy was negatively mentioned: *“I could overhear a conversation (not with another patient) while waiting in a changing area."; “I regularly overhear the discussions about the previously mentioned frustrations between receptionists—these are generally related to other members of staff and I do think this is unprofessional—you have a very busy waiting room and we are all hearing these comments. We all need to vent in our jobs at times, but there is a time and a place.”*

#### Lack of cleanliness

Others mentioned cleanliness, *“The toilets are not as clean and in good order as would be expected. I did not feel put at ease when I entered the building.”; “Washing hands before treatment, paper towels along the bed or something.”; “yes I was not happy having to wear a dressing gown used by previous clients without being laundered first”; “Reception process needs work—with a coffee machine essential in the reception area. Also—more importantly—the fact that I specifically highlighted several areas of potential bacterial growth within your treatment rooms, due to the physical design & build characteristics of specific items of equipment within those treatment rooms—YET MY COMMENTS WERE IGNORED BY A SENIOR STAFF MEMBER—is somewhat alarming in these days of more resistant bacterial infections…”.*

### Lack of comfort

#### Poor logistics

Poor logistics was a factor, including lack of car parking or receiving poor directions to the clinic: *“Parking is an issue.”: “Directions to the clinic.”*

#### Poor infrastructure

Poor infrastructure was mentioned as well: *“Perhaps for the future some temperature control in the treatment room.”; “The bench which patients lye is narrow, I am not a big man but I feel I am going to fall off. Most off putting and not a good thing if you are fearful of falling.”; “The building doesn't;t have disabled access to the room the chiropractor is in. It would be nice to be told if there is an alternative room on the ground floor.”; “A lick of paint here and there, primarily the changing cubicles.” O*thers thought furniture was not appropriate for older patients or people in pain: *“the chairs in reception are too low, and can be a little hard to get into and out off with a bad back”.*

#### Aesthetic/ancillary issues

Aesthetic issues were raised as well, with décor, available drinks, types of music, and general environment displeasing to a few: *“I feel the service provided is brilliant and would not change anything. I feel the waiting area is well lit and decorated with relaxing pictures. I feel it could benefit from a tv for patients to watch while waiting for treatments.”; “Improved reading material—probably a drinks machine—all satisfactory”; “Maybe quiet music in the care room as I was conscious my tummy kept rumbling!”; “room environment could be more conducive to relaxation”.*

#### Treatment attire

Treatment attire again was mentioned, but in a way we interpreted to relate more to convenience than safety:* “Gowns aren’t big enough and Velcro is old and doesn’t work very well.”*

### Lack of professionalism

#### Poor professional communication

Some participants believed that communication could have been better: *“More explanation as to what my practitioner is doing during the treatment”; “Written/illustrated exercise sheets would be useful a it is difficult to remember all instructions given at a session!”; Perhaps a written diagnosis or diagram showing areas of concern and how treatment will benefit me would be useful. Once back home, it was difficult to explain to family members what treatment I was having and why. Specific exercises to do at home may also be helpful.*

#### Poor professional procedures

Others thought procedures could be improved: *“Actual treatment/manipulation lasts just a few minutes. At the last visit I was told that the presumably out-of-place vertebrae had returned to its original wrong position. Was the treatment not adequate? Could/should I have been given directions as to what movements to do or to avoid? Any exercises at home?”; “In the first instance, the practitioner did not know my name, and after filling out a prior questionnaire with regard to general and medical history, i find a off putting from the start. i felt the initial session was being rushed and that time pressures were a concern for the practitioner.”; “Very short treatment time allowed.”*

## Discussion

This study used a qualitative approach, first prioritising responses with word frequency analysis, then content analysis to explore meaning and detail. First and second order themes were developed from codes for each of the responses containing the 20 most commonly encountered words in free text boxes completed by chiropractic patients on the Care Response database. The people who responded to the free text PREM questions in the Care Response system reported both positive and negative perceptions, which gave insight into the priorities of chiropractic patients. The 5 themes that emerged were satisfaction, value, safety, comfort, and professionalism.

### Good points: general

We found that the respondents in this study generally reported a positive perception of their experiences. This is consistent with other chiropractic studies on a variety of populations [18–21, 23, 24, 38–41]. In addition, Hurwitz in 2012 [42] reviewed the literature and found high satisfaction among patients of clinicians who employed spinal manipulation, including chiropractors. Specific first and second order themes will be explored below.

### Satisfaction

#### Satisfied with care

Respondents reported reduction in pain and improvements in function after treatment. This is consistent with other studies [20, 38, 43]. Herman [21], found high rates of satisfaction in chiropractic patients with chronic low back and neck pain with reasons including avoiding narcotics and surgery, which did not feature in our study. Alcantara [18] also reported that respondents to a survey rated their chiropractic care as “effective” or “very effective.”

#### Satisfied with staff

Regarding satisfaction with staff, we did not find these ideas specifically expressed in previously published studies, although Crowther [44] had similar but broader categories of “practitioner attributes” and “practice attributes.”

#### Unspecified satisfaction

In 2015, Houweling [23] used a Likert 5-point scale to measure satisfaction in Swiss patients with musculoskeletal problems and reported that respondents were more satisfied with chiropractic than medical care, but that there was no significant difference in patient global impression of change results. Crowther used a theme called “gestalt”, described as “a general sense of they were satisfied, or dissatisfied, with their health care professional based on overall, general actions of their practitioners on every visit” [44]. This seems similar to our “unspecified” theme. Crowther found no reports of gestalt satisfaction with chiropractors but some with dissatisfaction. The contrast with our findings may lie in Crowther’s definition including “every visit” but there could be other, unknown factors at work.

### Felt valued as a person

We separated feeling valued from other themes and included two second order themes under it: good logistical support and good emotional support. We did not specifically find the theme of feeling valued as a person in other studies. The ideas herein may have been categorised under professionalism or empathy or another theme by other authors.

### Safety

We only developed the theme of safety after coding the responses under areas for improvement. It may be that feeling safe was an assumption granted when visiting a chiropractor and therefore only worth raising as an issue if it was not present. However, we have no data on this. There may be other factors at work.

### Comfort

We developed a theme of comfort, comprised of physical and mental comfort second order themes, although we had not found this commonly in the published literature. Crowther [44] interviewed 197 Ontario (Canada) patients and reported greater breadth of categories of issues than most other studies. These included items such as office wait times, advocacy, and general practice attributes. Items were also reported positively or negatively, similar to Care Response. Crowther defined comfort as “limited parking, lack of wheelchair ramps, heavy doors that impeded access, lack of snow clearing, poor climate control, and absence of simple amenities such as coat racks.” [44] Most of these relate to physical comfort, whereas we coded them into logistics under the theme of feeling valued as a person or the theme of safety. Crowther [44] may have included some of these mental comfort themes under the category of “staff attributes” but it was not defined in the paper.

### Professionalism

Professionalism as a theme was divided into professional communication, procedural professionalism, and again, some participants did not specify professionalism. Communication was valued in relation to satisfaction in our study. This may be particularly important. In 2006, Gaumer [40] conducted a literature review and found satisfaction with care but inconsistent reasons for that satisfaction. However, he did find high correlations of satisfaction with good communication and empathy in the practitioner. Empathetic communication was also found to be valued by the participants receiving chiropractic care in a French hospital [20].

This aligns with the findings of Jensen [38], who reported that patients in Denmark appreciated a thorough examination by a chiropractor, and also advice and information on symptoms and prognosis. The setting of Denmark for Jensen’s study makes it somewhat unusual, though, in that chiropractic is integrated into the national health system there and funded by the government. This may have led to the development of themes that did not apply in the case of our study, including understanding standardised care packages within the Danish health system and appreciation for the high level of coordination between health care practitioners.

In addition, participants valued friendly, professional, and on-time service. It should be noted that we did not define professionalism here, as different respondents may have individual understandings of the term. Mallard’s [20] survey also found professionalism to be a positive factor in the patient experience.

### Areas for improvement: general

The negative experiences were reported as the opposite of the good points. That is, the data included reports that respondents sometimes felt rushed through treatment, that the treatment was not worth the cost, or that they weren’t treated professionally, empathetically, and with respect for them as individuals. Chou et al. [45] explored the perceived needs of patients seeking care for low back pain and found that practitioner confidence, communication correlated with patient satisfaction. They also found that dissatisfaction often arose from inadequate explanations of the problem and lengthy waiting times for referrals or appointments.

#### Areas for improvement: none

In the literature, we did not find reference to respondents indicating that they could cite no areas for improvement. It may be that authors did not include examples of people answering an “improvements”-type question in the negative. However, we decided that since respondents made the effort to express that they could think of no improvements to be made, it was worth reporting in this paper.

### Dissatisfaction

Issues related to care or cost were reported. The concern for cost contrasts with the findings of Weigel [22], who reported satisfaction with the cost of chiropractic care. However, that study focused on the United States of America, whereas Care Response data are international, and may reflect different payer schemes. Crowther [44] found that cost was neither a source of satisfaction nor dissatisfaction for Ontario chiropractic patients.

### Did not feel valued as a person – poor logistical support

Again, Crowther [44] had similar but broader categories of “practitioner attributes” and “practice attributes,” which were cited in both the positive and negative categories by participants.

### Lack of safety

We did not find issues of patient bodily safety (inappropriate clothing or audio privacy) or hygiene issues in published papers. However, they would seem to be worthy of the attention of clinicians and further exploration by researchers.

### Lack of comfort

Factors such as poor logistics or infrastructure, treatment attire in terms of convenience or aesthetic/ancillary issues may have been captured by Crowther [44], under “practice attributes” but no specific data were published with the paper. Otherwise, we found nothing in the literature, and believe that these issues are worthy of attention by clinicians as well as researchers as it seems that they do have importance, at least to some patients.

### Lack of professionalism

#### Poor professional communication

It is common knowledge that communication is crucial in clinical situations, and this second order theme was present in our responses. In 2022, Eindoven [41] qualitatively investigated the expectations and experiences of care provided by “sports chiropractors.” The study reported high levels of satisfaction, but that some respondents thought that professional communication could be improved.

#### Poor professional procedures

Comments relating to professional procedures often focused on time pressure. Patients reported that they did not appreciate being rushed or having short treatment appointments. Eindhoven [41] also found that some patients reported treatment times as too short.

In summary, free text comments entered by patients in an electronic PROM/PREM system appear useful in understanding their experiences with chiropractic care. Additionally, the insight this provides has the potential to provide a richness of detail which may be missed if only reviewing predetermined responses to specific questions set by clinicians/researchers.

### Methodological considerations

This is the first study to explore the free text responses of Care Response patients on their perceptions of their care. We analysed a large volume of data from international sources. We also interrogated it in more detail, developing more themes about patient perceptions of chiropractic care than most studies. The only reference to comfort we found in the literature was in one other study [44] and we found nothing on safety. We believe that these may be areas for further exploration. Using a qualitative approach to data analysis, as we have done in this study, potentially introduces bias from the authors [37]. However, bias may be mitigated to some degree by transparency, so that readers may better understand the perspective from which we interpreted the data, and the value judgments we might bring [37]. All 3 authors are registered chiropractic clinicians of over 20 years’ experience each. Therefore, we are “participant observers”, that is, we are investigating a system of which we are part. Although this status has the potential to introduce bias, it may also bring advantages such as knowledge of jargon and intra-professional issues. All 3 authors focus on musculoskeletal care (i.e. as opposed to a broad scope of practice); we all believe in an interdisciplinary approach to patient care, and we all value scientific evidence over deference to professional traditions. Therefore, we may have interpreted certain words or phrases and determined codes and themes differently than those with different experiences or who hold different values.

The use of CR is voluntary, so the sample group included in CR is limited to only those patients who chose to respond, and who attended clinics where the practitioners have chosen to enrol on the system. Consequently, the cohort would seem to include only motivated patients of motivated practitioners. Therefore, the potential bias of the sample is unknown. We may not have captured words if they were misspelled, leading to undercounting some responses. However, we believe this effect to be minimal. We may have interpreted words differently to the meaning intended by some respondents. As developing the codes was a manual process, it is possible that we missed information that appeared only a few times in this large amount of data. In using the word count method to sample the data we may have missed themes that were represented by a minority of respondents, and therefore not captured in the most common words. We did not compare the free-text data with quantitative data that the Care Response system also captures for participants and doing so may improve context and understanding of the free-text statements. We made no correlations between age, sex, or number of visits to a practice and any of the themes. This could be investigated in the future.

It would be useful to discover which codes were most frequent, to gain deeper insight into patient priorities for care. Additional studies could utilise interviews or focus groups to ensure accurate interpretation of meanings of patient responses to better provide safe, effective care. It would also be useful to further investigate the themes that appear only sparsely in the literature, such as comfort, safety, and aesthetic issues.

## Conclusions

The people who responded to the free text PREM questions in the Care Response system often reported positive experiences with their chiropractors, including that they had reduced pain, improved function, and felt validated in their clinical condition. In addition, they appreciated having diagnostic and treatment procedures explained to them. They valued friendly, professional, and on-time service. The negative experiences were reported as the opposite of these, when they felt rushed through treatment, that the treatment was not worth the cost, or that they weren’t treated professionally, empathetically, and with respect for them as individuals. A few reported safety, hygiene, or comfort issues, which we did not find in the literature. Chiropractors should note these priorities and consider engaging with patients according to them. Education institutions should consider how good practice and patient preferences in these areas might be incorporated into curricula.

### Supplementary Information

**Additional file 1.** Quotes representative of the different uses of each of the top 20 most common words entered into the “good points” and “improvements” boxes on the Care Response database.

## Data Availability

Data will not be available due to the proprietary interests of Clinical Transparency, Ltd.
