# Supplementary material for: A qualitative analysis of free-text patient satisfaction responses in Care Response, a database of patient-reported outcome and experience measures
Source: Chiropr Man Therap. 2024 Jan 29;32:2. doi: 10.1186/s12998-023-00528-7 (PMC10823652; doi:10.1186/s12998-023-00528-7)
Supplement: Supplementary file 1 — Additional file 1. Quotes representative of the different uses of each of the top 20 most common words entered into the “good points” and “improvements” boxes on the Care Response database. [file 12998_2023_528_MOESM1_ESM.docx]

**Additional file 1: Quotes representative of the use of each word**

**“Good points” words, one quote for each context (copied and pasted directly without editing for spelling, grammar, etc.)**

| **Word** | **Representative quotes** |
| --- | --- |
| Treatment | 1. It seems to be helping more than most other treatments I have tried 2. I received prompt treatment and a better understanding of my problem. After my course of treatment I felt healthier, pain free and generally more comfortable. 3. A good chiropractor who listens to ones problems and discusses any treatment needed. 4. Feel confident that I have a well qualified and experienced Chiropractor. Good explanations about each element of treatment, its purpose and what impact I will feel in the treatment area. 5. i feel at ease and not worried a bout the treatment im about to recieve as im well informed and made to feel relaxed 6. Extremely professional practice and the one chiropractor I have had treatment from. Gave me great confidence in the treatment I would receive. |
| Feel (-ing) | 1. really knows what he's talking about and I always feel listened to 2. I feel in very safe hands and will receive good advice. 3. Always has time to listen to your concerns, never rushes you, caring and thoughtful. Makes you feel at ease with the session. 4. I was so upset, worried and feeling very down on my first visit to the chiropractor. I was immediately put at ease and listened to and I feel like a different person and do more now than I could before and it has made me feel positive and happy again. I cannot thank you enough for the wonderful treatment and service I have received. 5. Everything well explained and feel better after my visit 6. Very good, not made to feel rushed in any way, relaxing and informative. 7. He explained what he was going to do & how it should feel after. |
| Good | 1. Really good clinic, excellent care 2. The variety of methods available and used to treat my problems. Listens to what I say and gives good advice. 3. Good explanation of aims and objectives of care. Support and advice outside of appointment when neeeded. 4. It was good that I rang up with a problem I discussed with my chiropractor and got an appointment on the same day with my usual chiropractor. 5. Mostly on time, understand the issues and provide good treatment. 6. He seemed to be very good in general. Good knowledge about back problems. 7. Local practice with plenty of free parking. No waiting times. Good reception & expert chiropractor. 8. So accommodating to offer early appointments for patients. Chiropractor had a good sense of humour which aids relaxation and makes me feel at ease in his care. 9. Prompt service, good listener and explains well about care and exercises to do at home |
| Problem(s) | 1. Treatment was very good and resolved 90% of the problem on one visit. Final visit cured all. 2. explanation of what had happened and suggestions as to how to deal with back problem should it recur. 3. He makes me feel that my problem is important and he really wants to help me. 4. The right questions are asked to get to the root of the problem and then treatment is efficient and effective. 5. The cause of my problem was very quickly diagnosed and explained and a treatment plan produced. |
| Friendly | 1. Friendly, helpful kind and considerate. 2. Friendly, professional, competent which prvodies reassurance to me as the patient that my chirpractor actually cares about what he does. 3. Both the Chiropractor and Receptionist are friendly, professional and helpful. 4. Excellent service. Very friendly clinic, brilliant receptionist. |
| Care | 1. They do care, they respond quickly and help get you on a road to recovery. 2. Really good clinic, excellent care 3. explaining and informing client about there care each step of the way, don't loose this. being treated as an individual. 4. Impressively flexible care coupled with sensitive adaptability to resolving my source of pain, plus a regular infusion of hope :) 5. The care is excellent at every stage so far. My chiropractor is so personable and seems to genuinely care about my problem and wants to help to make it better. 6. Very professional care right from reception throught to treatment 7. Very profe3ssional and knowledgeable. Personalised care and advise. |
| Chiropractor | 1. Friendly, informative and patient chiropractor. 2. Very knowledgeable chiropractor and helpful 3. The chiropractor listens to my problems and explains what he can do to help 4. very patient and professional chiropractor and has certainly helped me deal with my problems with my back. 5. The chiropractor is empathetic which is a great help to ease worries , as pain creates fear for the future wellbeing. 6. Brilliant chiropractor and staff 7. Her approach to her patients is very gentle and down to earth. |
| Ease(s) | 1. Always has time to listen to your concerns, never rushes you, caring and thoughtful. Makes you feel at ease with the session. 2. Takes time to explain how I can ease the pain between visits ie. Stretches and exercises |
| Excellent | 1. Excellent manner and good practical advice. Very impressed. 2. Excellent care 3. It is excellent 4. Excellent service, have recommended to friends and colleagues 5. Excellent staff, kind, polite, very obliging 6. I find her to be a very natural and down to earth person and above all an excellent listener with good knowledge, oh and funny. Despite the depression I have, she cheers me up when I visit. 7. Excellent explanations of what is being dome and why, and of problems themselves. 8. Very professional chiropractor. Excellent advice always given to me. Excellent duty of care to her patients. 9. Overall excellent experience and excellent results from treatment |
| Professional | 1. Clean and professional 2. Patient centered planning is an obvious and positive part of the procedure delivered in a professional and friendly manner. 3. All the staff I have met have been friendly, helpful, knowledgeable and professional, could't really ask for more. 4. First class professional service that I would highly recommend to anyone. 5. Caring, friendly but professional manner |
| Pain | 1. Advice and treatment have lessened frequency and intensity of lower back pain - a good result. 2. Very professional and caring individual, who takes the time to explain how different muscles work in the back, shoulders and neck and how they transfer pain. 3. She is caring and understood the amount of pain I was in at the beginning of my treatment |
| Caring | 1. Caring and efficient. 2. Always has time to listen to your concerns, never rushes you, caring and thoughtful. Makes you feel at ease with the session. 3. all staff and chiropractor very pleasant and caring |
| Time(s) | 1. He is lovely and will always take the time to listen to me and explain things if I have any questions or concerns. He is very patient and easy to talk to and get along with. 2. Very flexible appointment times, and my usual chiropractor is very approachable and makes me feel at ease. 3. Greatly appreciate being seen on time. Calm and friendly environment. |
| Understanding | 1. very understanding feel reassured 2. She always makes you feel welcome and is very friendly, she also goes that extra while with understanding my working background and has tried to bare that in mind and give me advice in ways to help. 3. Great service, very understanding chiropractor who is trying very hard indeed to resolve my problems. 4. Very sypathetic understanding staff |
| Thorough | 1. Never feel rushed, always a very thorough examination. Always feel better when finish a session and have discussed timing of next visit. 2. Thorough explanation when discussing my treatment and always ensures I'm comfortable when I leave. Demonstrating the exercises/stretches given is very useful. 3. Very thorough and thoughtful care |
| Always | 1. chiropractor very good and always listens. 2. Always explains how problem being treated, follow up exercises & advice 3. Very easy to talk to, always feel very relaxed around her. 4. Always professional, friendly, understanding, and considerate. Very willing to answer my questions to help me understand my condition, and always gives me much needed encouragement to aid my own efforts to overcome my extremely painful but temporary disability. 5. I nearly always feel better immediately. I don’t very often need to return, things settle down fairly promptly. 6. very well run service, appointments are always on time, professional but welcoming 7. Excellent communication throughout the treatment. Understanding and very approachable approach to care. Always friendly positive and a pleasure to be cared for by. |
| Back | 1. Yes, helped so much my back pain 2. very thorough in explaining the reasons behind the issues I was having with my back |
| Everything | 1. I have learned a lot about the set-up of the body. Everything has been explained clearly and in terms that I understand. I am now a lot more informed about my issues and care. 2. Everything really, the clinic is always warm which is a big help. 3. Listens to everything I have to say and answers all my questions. 4. Thoroughness with which he checks every aspect of how my body is working. Amount of time he spends making sure everything is right. Encouraging and friendly approach. Explained the problems I had in detail the first time I went there, which I really appreciated. |
| Service | 1. Good all round service 2. Small clinic, so feeling of personal service. Later appointments good. Practitioner friendly and approachable. Good quality care. 3. Very friendly and relaxed service and the treatment is helping me to get better. 4. Excellent professional service- clear instructions- first time ever needed service 5. Very prompt service, knowledgeable staff, friendly office, and convenient location and scheduling. |
| Explains | 1. Listens to the symptoms. asks relevant questions, explains the problem(s). Suggests exercises to speed recovery. 2. Chiropractic is patient and explains the symptoms and treatment being undertaken 3. he listens, explains everything and is very thorough |

**“Improvement” words (copied and pasted directly without editing for spelling, grammar, etc.)**

| **Word** | **Representative quotes** |
| --- | --- |
| Nothing/anything | 1. Nothing 2. Nothing at the moment 3. Have nothing to compare it with, but cannot see any areas needing improvement. |
| Appointment(s)/ session(s) | 1. Appointments after the initial consultation could be just a little longer than they currently are (15 mins) 2. More late appointments would be beneficial. 3. Shorter waiting times. I always had morning appointments ( varying from 9.45 to 11am ) and I had to wait up to 40 minutes past my appointment time. 4. Maybe text appointment reminders 5. availability on nhs - I struggle to cover costs |
| Think | 1. Not that I can think of. 2. I think a 20 minute appointment is a bit tight, timewise, both in terms of discussing how things are going and getting some treatment done. 3. I think there could have been a more thorough enquiry into my overall health other than just a questionaire. |
| Can/Can’t | 1. nothing I can think of 2. Nothing can be improved. |
| Time(s) | 1. a little more time for treatment sometimes seems rushed 2. Waiting time in reception could be improved. Experienced delays with previous patient hold ups and then when being attended to myself my appointment time appears to have been shortened in order for back log to catch up. |
| N/A/none | 1. None |
| Treatment | 1. Perhaps a written diagnosis or diagram showing areas of concern and how treatment will benefit me would be useful. Once back home, it was difficult to explain to family members what treatment I was having and why. Specific exercises to do at home may also be helpful. 2. The cost was very high considering my seoncd treatment was less than ten minutes. This was disproportionate. 3. the lenghth of the appointments is problematic - 15 minutes per session doesn't give nearly enough time for discussion, explanation, reflection, planning , as well as physical treatment - 4. No, very impressed with my care and treatment 5. Would like to see treatment on the NHS. |
| Happy/good/ excellent/satisfied | 1. Not that i can think of. Very happy with the clinic and would recommend. |
| Longer | 1. perhaps the treatment could last a little longer |
| Feel | 1. Not at the moment, I feel at this early stage of my care, the practice is performing brilliantly. 2. I find timing a little stressful. I suppose extending the length of sessions isn't possible but I do feel a bit rushed sometimes. 3. To explain a little more how working on one area is helping another, as I can sometimes feel my real problem areas are being overlooked, but it’s most likely another area is directly affecting these areas 4. The room feels too bare, a bit too clinical. |
| Chiropractor | 1. Maybe longer time/manipulation with chiropractor 2. Just the time inbetween getting undressed and being seen by chiropractor did have to wait a little while. 3. Lacks empathy; don't feel any connection. Chiropractor is completely competent but does not connect with me. 4. Chiropractor does not explain what they are doing, it would be helpful to know. Some treatment is very painful. |
| Little or bit | 1. Sometimes the session felt a little rushed, it is only 15 mins but as I was a lot better felt like the session didn't fill the time slot. When you're paying privately you want your money's worth! 2. Waiting time for my first appointment was about 20 mins - a little longer than expected. 3. Changing facilities could be better, nicer decor and a little more private 4. I would like a little more information/description of my problem and how long it may take to recover (if that is possible) |
| Service(s) | 1. Can't fault it, excellent service |
| Wait(-ing) | 1. Often they are running quite late so there can be a wait 2. The waiting area is a little small & dingy but there are never too many people in there at the same time to mean you dont have a seat or are all squashed in. 3. The time you have to wait for another appointment as they are booked up very quickly 4. In some rooms when you have stripped off and are sat on the bed waiting for the chiropractor to come back into the room you are sat directly In front of the door - I felt a bit awkward |
| Room | 1. Rooms could be warmer 2. some waiting room seats could have arms to help you get up if you have a bad back 3. Consultation room not sound-proofed - can hear other patients while waiting for my own appointment so assume I can be heard too. 4. Less time sitting in the waitign room as my time is important as well. One hour waiting is unacceptable 5. Remve the radio from the waiting room |
| Exercise(s) | 1. Advice how I can maintain the ongoing improvements (e.g. exercise) 2. Written/illustrated exercise sheets would be useful a it is difficult to remember all instructions given at a session! |
| Clinic | 1. Nothing I can suggest, I think your Clinic is first class all round. 2. a ground floor clinic so less mobile people can enter more easily 3. The clinic could be open later in the evenings, as many people can not get time off work. The price of each session could be a bit cheaper, especially if you do many sessions, it works out quite expensive. I did not beforehand how many sessions I would need in total; I understand this as each individual heals in different times, but it would be easier to have an estimate in order to plan my expenses for each session. 4. Always feels quite rushed but I appreciate that the clinic is busy |
| Back | 1. The gowns that you change into are really hard to fasten at the back yourself - especially when you probably can't really move as well as normal due to your condition. |
| Massage | 1. A little bit more time loosening of muscles before treatment. Also massage after treatment would be good too. |
| Gown(s) | 1. The size and quality of the gowns! 2. When dressed in gown I feel slightly uncomfortable sitting back in waiting room wearing this. 3. May wear leggings and vest instead of the gowns |
